# Latest development in microalgae-biofuel production with nano-additives

**DOI:** 10.1186/s13068-019-1465-0

**Published:** 2019-05-20

**Authors:** Nazia Hossain, T. M. I. Mahlia, R. Saidur

**Affiliations:** 1 Department of Civil and Infrastructure Engineering, School of Engineering, RMIT University, Melbourne, VIC 3001 Australia; 2 School of Information, Systems and Modeling, Faculty of Engineering and Information Technology, University of Technology Sydney, Sydney, NSW 2007 Australia; 3 Research Centre for Nano Materials and Energy Technology (RCNMET), School of Science and Technology, Sunway University, No. 5, Jalan University, 47500 Bandar Sunway, Petaling Jaya Malaysia; 4 Department of Engineering, Lancaster University, Lancaster, LA1 4YW UK

**Keywords:** Microalgae, Microalgal biofuel, Nano-additives, Bioenergy, Biodiesel

## Abstract

**Background:**

Microalgae have been experimented as a potential feedstock for biofuel generation in current era owing to its’ rich energy content, inflated growth rate, inexpensive culture approaches, the notable capacity of CO_2_ fixation, and O_2_ addition to the environment. Currently, research is ongoing towards the advancement of microalgal-biofuel technologies. The nano-additive application has been appeared as a prominent innovation to meet this phenomenon.

**Main text:**

The main objective of this study was to delineate the synergistic impact of microalgal biofuel integrated with nano-additive applications. Numerous nano-additives such as nano-fibres, nano-particles, nano-tubes, nano-sheets, nano-droplets, and other nano-structures’ applications have been reviewed in this study to facilitate microalgae growth to biofuel utilization. The present paper was intended to comprehensively review the nano-particles preparing techniques for microalgae cultivation and harvesting, biofuel extraction, and application of microalgae-biofuel nano-particles blends. Prospects of solid nano-additives and nano-fluid applications in the future on microalgae production, microalgae biomass conversion to biofuels as well as enhancement of biofuel combustion for revolutionary advancement in biofuel technology have been demonstrated elaborately by this review. This study also highlighted the potential biofuels from microalgae, numerous technologies, and conversion processes. Along with that, the study recounted suitability of potential microalgae candidates with an integrated design generating value-added co-products besides biofuel production.

**Conclusions:**

Nano-additive applications at different stages from microalgae culture to end-product utilization presented strong possibility in mercantile approach as well as positive impact on the environment along with valuable co-products generation into the near future.
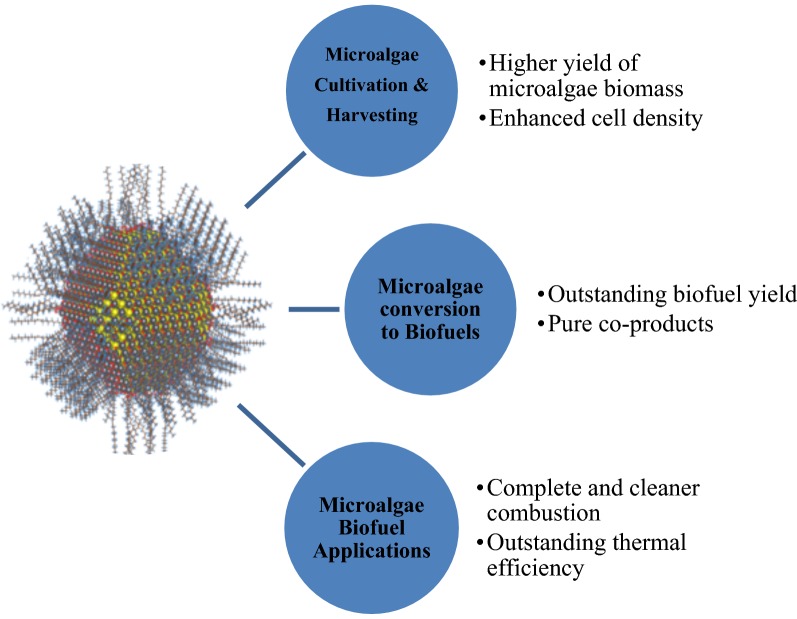

## Background

**Fig. 1:**
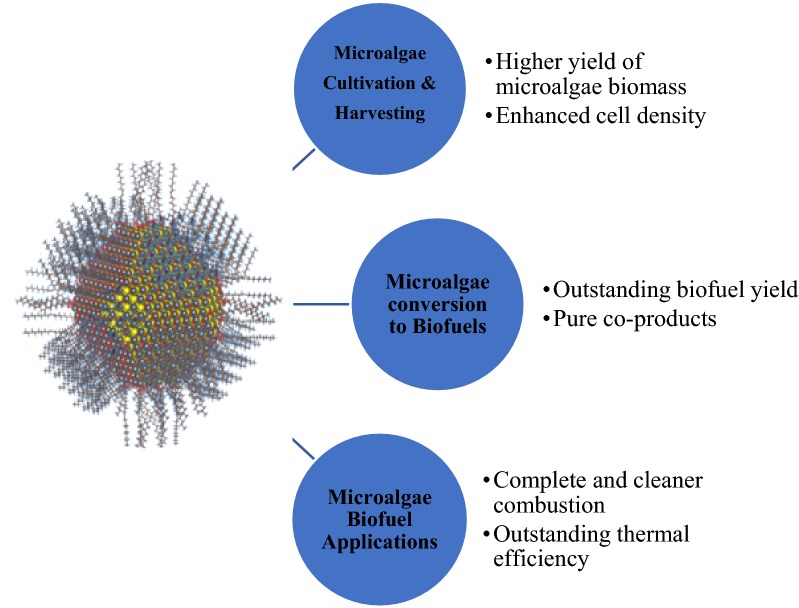
Nano-additive applications for the enhancement of microalgae cultivation to biofuel implementation

Biofuel has caught substantial attention worldwide nowadays as an alternative fuel due to its capability to adapt with gasoline for a maximum 85% blend without any engine modification. Subsequently, the suitability of various candidates for biofuel is being continuously quested by the researchers and environmentalists [1–5]. In this recent era, one of the most sophisticated technologies, nano-technology integration with bioenergy application by the nano-energy sector has brought a revolutionary impact on biofuel conversion processes and enhancement of engine performances. Nano-technology is defined as designing a device or material in nano-scale (10^−9^ m). To accelerate the biofuel yield and improve the efficiency of biofuel utilization in petrol and diesel, nano-technology has been initiated via nano-additives such as nano-magnets, nano-crystals, nano-fibres, nano-droplets, and others [6–8]. Figure 1 presents the perspectives of nano-additives on microalgae cultivation to microalgal-biofuel implementation.

On this eve of the quest for suitable biomass for biofuel, the concept of microalgae cultivation appeared to the spotlight for biofuel manufacturing due to several positive perspectives such as (i) they do not clash with human or animal food chains, (ii) very rich with carbohydrate, protein, and oil content, (iii) can grow in aqueous media such as wastewater, freshwater, saline water, and assimilate nutrients from brackish water, salt water, or highly polluted water, (iv) demand low water, (v) sustain capability to grow whole year naturally with sunlight presence, (vi) can be cultivated in the waste dump area, sea, ponds, rivers, industrial, and municipal waste drainage, wet bare lands especially in cold regions, (vii) develop sustainable O_2_ generation system, and (viii) diminish CO_2_ by up taking it for photosynthesis respiration [9–14]. In addition, microalgae contain very short harvesting life cycle and yield nascent biomass that drives higher productivity of the desired biofuel. Interestingly, microalgae carry a prodigious amount of carbohydrates, protein and lipid, the sole components of biofuel conversion [13, 15–17]. Nano-technology applications have been implemented to biofuel industries, since the existing controversial approaches of traditional microalgae culture-biofuel production contain a number of limitations such as inconsistent industrial-scale microalgae production, high microalgae production and harvesting cost, energy consumption for biofuel production from microalgae, and the increase of greenhouse gas intensity in environmental [18]. Nano-technology applications can be entailed in different stages from microalgae cultivation to microalgae-biofuel application in fuel engines due to durability, recyclability, adsorption efficiency, catalytic performance, stability, crystallinity, economical advantage, high storage capacity, excellent biofuel yield, and environment-friendly characteristics. According to the previous studies, nano-technology application enhanced microalgae cultivation, the maximum yield of numerous microalgae biofuels as well as microalgae-biofuel implications in petrol and diesel engines. Various nano-materials, e.g., nano-fibres, nano-particles, nano-tubes, nano-sheets, and other nano-structures, have been investigated as effective nano-catalysts in direct and indirect approaches in biofuel (e.g., bioethanol, biodiesel, biomethane, and others) yield enhancement [19–22]. For instance, magnetic nano-particles were used as a carrier for enzyme immobilization for bioethanol and biodiesel generation effectively. Owing to high coercivity and powerful paramagnetic characteristics, magnetic nano-particles were also preferred for methanogenesis to produce biomethane [21].

To authors’ best knowledge, no review study has been performed on numerous biofuel productions from microalgae integrated with the nano-additive application so far. The closest review with this study was conducted on the bioenergy production from lignocellulosic biomass (agricultural residues), industrial waste (sludge) as well as algae (microalgae and macroalgae) with the nano-scale optimization which has merely emphasized on the mechanism of nano-particles, biomass characteristics, and nano-particle application on biomass growth [23]. Compared with that, the current review contextualized the numerous biofuel productions from pure microalgae and optimization with nano-additive application on biomass growth to end-product application. Therefore, the major objectives of this review work are (i) to determine the array of the techniques and methods associated with nano-particles incorporation with microalgae culture as well as microalgal biofuel, (ii) to demonstrate divergent nano-additive applications on microalgae cultivation, biomass conversion to biofuels, and biofuel combustion, (iii) to identify the potential sources of microalgae, especially the carbohydrate, protein, and lipid-enriched microalgae types for biofuel production and determine the possible microalgae biofuels, biomass conversion technologies, and processes to biofuels, and (iv) to assess the future prospects of the process development planning along with integrated design of some other value-added products besides biofuel.

### General perspective of microalgae

Microalgae are referred as photosynthetically driven single or multi-cellular living being, the habitat of moist environment either on the solid mud or float on various water types, e.g., fresh water, marine water, wastewater with the presence of sunlight, or artificial light. The scientific consensus is that through photosynthesis respiration, they convert CO_2_ to O_2_ and generate large amounts of cellular energy content embedded with sugar, protein, and lipid [24–27].

Nowadays, industrialization and urbanization threaten the existing ecosystem severely by dumping heavy metal waste containing phosphorus, nitrogen, sulfur and others as well as exhaling high amount CO_2_ to the free air. Another knocking threat to the energy sector is rapid depletion of fossil fuel worldwide due to excessive energy uses [28–30]. With this circumstance, microalgae cultivation in the wastewater, unused fresh, and saline water, drainage is considered as suitable scientific solution for green energy due to some favorable aspects such as multi-functionality, genuine conversion competency biologically and flexibility with growth system, wastewater accumulation, CO_2_ sequestration, and large amount of carbohydrate–lipid–protein content. To note, carbohydrate–lipid–protein are the main components to generate divergent biofuels (e.g., bio-oil, biodiesel, biobutanol, and others) and biogas (e.g., bio-hydrogen) [31, 32]. The cellular components of microalgae are composed of huge fraction of lipid, protein, and carbohydrates resulting in the driving factors of biofuel production. Table 1 presents some well-known potential microalgae candidates for biofuels. These species were extensively researched in the laboratory and large-scale applications so far. Type and description of these species have been tabulated to present a detailed view of selected species, suitable growth conditions (such as water type and region for cultivation), availability, and cellular specifications. Table 2 represents prime microalgae cellular component composition of several well-known microalgae species for biofuel production [24, 33, 34].

**Table 1:** Several potential microalgae species for biofuel production, type, and description of the species in brief [35–42]

Microalgae name	Type	Description in brief
*Chlamydomonas reinhardtii*	Chlorophyta	Genetically modified by sex-cross, contains high amount of carbohydrate, lipid and protein in cell wall
*Chlorella* sp.	Chlorophyta	Unicellular green microalgae, source availability of tropical water with enough solar light
*Spirulina platensis*	Chlorophyta	Spiral-shaped multi-cellular microalgae (with no true nucleus), fresh water habitant, contains lipopolysaccharides and peptidoglycan (carbohydrate components) in cell wall as well as cyanophycean and starch are the main carbohydrate storage products
*Chlorella vulgaris*	Chlorophyta	Spherical shaped, single cellular (with nucleus) microalgae, grows in both fresh and marine water with adequate sunlight, contains cellulose and hemicelluloses (carbohydrate components) in cell wall and starch is the main carbohydrate storage product
*Botryococcus braunii*	Chlorophyta	Green microalgae, shape type pyramidal, source availability of tropical and oligotrophic freshwater such as lakes, ponds, estuaries
*Ostreococcus tauri*	Chlorophyta	Eukaryote and unicellular
*Phaeodactylum tricornutum*	Phaeodactylum	Salt water diatoms
*Nannochloropsis* sp.	Heterokont	Grown in both saline and fresh water, genetically modified and paralleled recombinant microalgae type
*Symbiodinium* sp.	Fungia repanda	Source availability in sea water, advanced eukaryotic, dinoflagellates
Phytoplanktons	Either prokaryotic or eukaryotic	Usually autotrophic, source availability at saline and tropical water sources such as lakes, ponds with sufficient solar energy
Cyanobacterial Mats	Prokaryotic	Easily grown in saline water
*Saccharina japonica*	Brown type of microalgae	Grown at sea and coastal water sources
*Chlorococum* sp. and *Spirogyra* sp.	Cyanobacteria/blue–green algae	Shape type spiral, source availability in usually moist environment, marine and fresh water sources, grown randomly in tropical areas where sunlight is available sufficiently

**Table 2:** Approximate carbohydrate–protein–lipid–ash content composition of suitable microalgae species (dry weight) for biofuel production [32, 43–45]

Type of microalgae	Total sugars (%)	Protein (%)	Lipids (%)	Others (including ash content) %
*Chlamydomonas reinhardtii*	48	17	21	14
*Chlorella* sp.	56	22	19	3
*Spirogyra* sp.	20	55	16	9
*Porphyridium cruentum*	35	50	11	4
*Spirulina platensis*	60	12	8	20
*Dunaliella salina*	57	32	6	5
*Bellerochea* sp.	3	24	15	3
*Chaetoceros* sp.	2	18	18	3
*Rhodomonas* sp.	9	74	15	2
*Scenedesmus* sp.	18	56	12	–

### Biofuels from microalgae

**Fig. 2:**
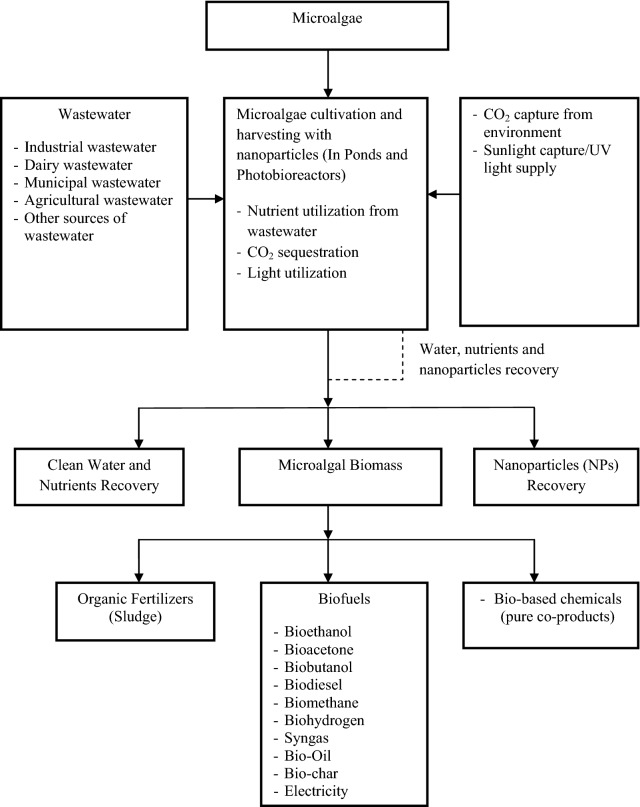
Process flow diagram of carbon capture, water treatment and biofuels production from microalgae incorporated with nano-particles [18, 25, 49–54]

Numerous biofuels, e.g., bioethanol, biodiesel, bio-oil, biomethane, bio-hydrogen, and others, have been extracted from microalgae [46, 47]. Nano-particles’ incorporation with microalgae cultivation (e.g., cell suspension, cell separation, and cell harvesting), biofuel conversion technologies, and biofuel application have amplified the overall yield in every stage [22]. According to the previous studies, a very small amount of colloidal hydrous iron(III) oxide particles boosted almost 100% microalgae cell suspension; magnetic particles incorporated with aluminum sulfate were very effective for cell separation from the mixed culture of *Anabaena* and *Aphanizomenon* microalgae species; silver nano-particles application on *Chlamydomonas reinhardtii* and *Cyanothece* 51142 microalgae harvesting increased 30% higher biomass productivity; and calcium-oxide nano-particles escalated the large-scale biodiesel conversion yield up to 91% via catalytic transesterification [18, 22, 48]. This study summarized the overall microalgae cultivation integrated with nano-particles until biofuel production in Fig. 2. Different biofuels from microalgae and conversion processes are diagrammed in Table 3.

**Table 3:** Possible biofuels from microalgae and conversion processes

Biofuel	Conversion processes	Some potential nano-additives for biofuel yield enhancement	References
Bioethanol, biobutanol, biomethanol, bioaceton	Simultaneous saccharification and fermentation (SSF) Separate hydrolysis and fermentation (SHF) Simultaneous saccharification and co-fermentation (SSCF) Separate hydrolysis and co-fermentation (SHCF) Dark fermentation Photo fermentation Anaerobic fermentation Acetone–butanol–ethanol (ABE) fermentation Consolidated bioprocessing	Fe_2_O_3_, CeO_2_-CNT and others	[42, 49, 85–89]
Biodiesel	Lipid hydrolysis and chemical, physical, enzymatic transesterification Interesterification Acidolysis Alcoholysis associated with glycerolysis Folch method (use of chloroform–methanol) Bligh and dyer method (use of chloroform–methanol) Modified method of the Folch/Bligh and dyer methods (use of methyl-tert-butyl-ether) Superior solvent extraction method (use of chloroform) Direct or in situ supercritical transesterification Post-transesterification Wet extraction method Deep eutectic solvent (DES) method	ZrO_2_, TiO_2_, Al_2_O_3_, CeO_2_, SiO_2_, Fe_2_O_3_, CaO-NPs and others	[46, 77, 78, 90–100]
Syngas	Gasification Pyrolysis Direct combustion Bio-electrochemical fuel cells Supercritical water gasification (SCWG) Hydrothermal gasification	TiO_2_, CeO_2_ and others	[88, 101–105]
Bio-electricity	Bio-electrochemical system contain anode, cathode and electrode Direct combustion	Fe_2_O_3_, CeO_2_-CNT, CaO-NPs and others	[106–108]
Biomethane	Anaerobic digestion Catalytic hydrothermal gasification	SiO_2_, nano-particles of platinum (Pt), nickel (Ni), cobalt (Co), iron (Fe) and others	[46, 109–112]
Bio-hydrogen	Solid-state anaerobic digestion Biophotolysis Photobiological hydrogen production Dark fermentation Photo fermentation Solid-state fermentation Suspended fermentation	TiO_2_, CeO_2_ and others	[113–118]
Bio-oil and bio-char	Hydrothermal (thermochemical) liquefaction Pyrolysis Expeller press Bead beating Ultrasound assisted extraction method Microwave extraction method Electroporation/electropermeabilization Slow pyrolysis Fast pyrolysis Torrefaction Hydrothermal carbonization Hydrothermal liquefaction Ion exchange Wet imprgenation Catalytic hydropyrolysis Hydrogenation Hydrodeoxygenation	CeO_2_-CNT, SiO_2_-MgO nanohybrids and others	[46, 90, 119–127]

## Preparing techniques of nano-additives for microalgae biofuel

Magnetic nano-particle (NP) powder has been enumerated to the microalgae cell suspension in the photobioreactor cultivation process to flocculate cells for uniform distribution of nutrients and light all over the reactor. Immunomagnetic detection and modification of microalgae cell by NPs are another well-practiced method for cell suspension enhancement. Nano-liquid has been injected to the cell culture for microalgae harvesting and bio-separation through this technique. Silver nano-materials have also been implemented on the photobioreactor surface coating for higher light accessibility [22]. Along with microalgae culture and harvesting, sphere nano-particles have been enacted during hydrolysis, lipid extraction, transesterification, and biofuel purification from microalgae via irradiation and ultra-sonication methods and much higher biofuel yield have been obtained. Another established method of nano-materials application includes enzymatic nano-catalyst, lipase carrier. The reactant diffusion rate enhancement by the NPs to the active side of lipase has been determined by Eq. 1 [55]:

1 $$R_{\text{df}} \propto \frac{1}{{D^{2} }},$$

where *R*_df_ = diffusion rate to the active sides of lipase and *D* = diffusion path diameter of reactant to the access of lipase active side.

NPs for biofuel doping can be formulated by either physical or chemical methods. For instance, plasma-arcing, sol–gel method has been presented as chemical method and ball mill process (agitation rate: 450 rpm) was presented as a physical method for NPs’ preparation in the previous studies [56–58]. Subsequently, NPs were doped with microalgal biofuel (e.g., microalgae oil, biodiesel, bioethanol, and others) with different doses (e.g., 25 ppm, 50 ppm, 100 ppm, and others) via ultra-sonication processing by the presence of magnetic stirrer and implemented on compression ignition (CI), direct ignition (DI) engines without any engine modification. NPs are dispersed in a base fuel and smoothen potential agglomerate into nano-scale due to its’ larger surface area and surface energy [58–61]. The ultra-sonication method was conducted with various parameters such as frequency (e.g., 20 kHz, 40 kHz, and 45 kHz), power (e.g., 120 W and 220 W), and time (30 min and 60 min) [60, 62, 63]. Cationic surfactants, e.g., tetra methyl ammonium hydroxide, cetyl trimethyl ammonium bromide, have been incorporated on the nano-particle surface for a negative-charge envelope formation to resist NPs’ sedimentation [56, 64]. After biofuel-NPs’ doping, the NP-blended biofuel was preserved under the static condition to stabilize for fuel purpose [59]. Several potential NPs–microalgae-biofuel blends are tabulated in Table 4. The morphology and crystalline phases after NP-doping were analysed through a scanning electron microscope and X-ray diffraction, respectively [61]. *Botryococcus braunii* oil was doped with almost 50 nm sized titanium dioxide (TiO_2_) and silicon dioxide (SiO_2_) incorporated with biodiesel (B20) of different doses for enhanced fuel efficiency in CI engine [60]. *Caulerpa racemosa* green algae biodiesel (B20) was doped with 50 nm sized zirconium dioxide (ZrO_2_) by the different doses for CI engine [59].

**Table 4:** Applications of different dosages of nano-particles on biofuels and combinations of biofuel nano-particle blend and application output

Microalgal and other biofuel	Sources for biofuel extraction	Nano-particles (NPs)	Dosage	Combinations of biofuel and nano-particle blends	Application output	References
Biodiesel	*Caulerpa racemosa*	ZrO_2_	50 ppm 100 ppm	B2050 ppm B20100 ppm	Reduction of hydrocarbon (HC), carbon-mono oxide (CO) Nitrogen oxides (NO_*x*_) emission increase	[59]
Biodiesel	*Madhuca longifolia*	TiO_2_	100 ppm 200 ppm	BD100T100 ppm BD100T200 ppm	Reduction of 5.8% unburned HC, 9.3% CO, 2.7% smoke and 6.6% NO_*x*_ emission	[128]
Biodiesel blended with diesel	*Jatropha curcas*	Al_2_O_3_ CeO_2_	30 ppm 30 ppm	B20A30C30 ppm	12% improved brake thermal efficiency Reduction of 30% NO_*x*_, 60% CO, 44% HC and 38% smoke	[61]
Biodiesel	*Jatropha curcas*	Al_2_O_3_ CeO_2_	30 ppm 30 ppm	B100A30C30 ppm	Improved brake thermal efficiency Reduction of NO_*x*_, CO, HC and smoke	[61]
Biodiesel	*Botryococcus braunii*	TiO_2_ SiO_2_	50 ppm 100 ppm	B20TiO_2_SiO_2_50 ppm B20TiO_2_SiO_2_100 ppm	Increased calorific value Decrease in brake-specific fuel consumption (BSFC) Improved brake thermal efficiency (BTE) Reduction of ignition delay time Improved brake thermal efficiency Improvement of combustion characteristics Minimum CO, HC Maximum NO_*x*_, CO_2_	[60]
Biodiesel	*Pongamia pinnata*	Rh_2_O_3_	100 nm	B100Rh_2_O_3_	Reduces CO, 37% NO_*x*_, 45% unburnt HC Improvement of thermal efficiency	[58]
Biodiesel	*Glycine max*	Co_3_O_4_	100 mg/l 38–70 nm	B100Co_3_O_4_	1.03% better engine performance than usual biodiesel combustion Reduction of smoke and 7.46% NO_*x*_ emission	[56]
Biodiesel	*Glycine max*	Al–Mg	100 mg/l 38–70 nm	B100Al-Mg	Better engine performance than usual biodiesel combustion Reduction of smoke and 16.33% NO_*x*_ emission	[56]
Biodiesel	*Jatropha curcas*	Al_2_O_3_ Al_2_O_3_ Carbon nano-tube (CNT) Al_2_O_3_CNT	25 ppm 50 ppm	BAl_2_O_3_ ppm BAl_2_O_3_ ppm BCNT25 ppm BCNT50 ppm BAl_2_O_3_CNT 25 ppm	Considerable enhancement of brake thermal efficiency Marginal reduction of harmful emissions Improved heat transfer rate Short ignition delay effect Enhancement of heat conduction properties and surface area/volume ratio	[129]
Biodiesel	*Azadirachta indica*	Ag_2_O	5 ppm	B100Ag_2_O 5 ppm	Decrease of 12.22% CO, 10.89% HC, 4.24% NO_x_ and 6.61% smoke Enhancement of brake thermal efficiency with reduction in brake-specific fuel consumption	[63]
Biodiesel	*Azadirachta indica*	Ag_2_O	10 ppm	B100Ag_2_O 10 ppm	Reduction of 16.47% CO, 14.21% HC, 6.66% NO_*x*_ and 8.34% smoke Significant improvement of brake thermal efficiency with reduction in brake-specific fuel consumption	[63]
Biodiesel	*Jatropha curcas*	Co_3_O_4_	–	B10Co_3_O_4_ B20Co_3_O_4_ B100 Co_3_O_4_	Reduction of the ignition delay Improvement of combustion by its’ catalytic effect Burning of the carbon deposits Reduction of black smoke	[57]
Biodiesel–bioethanol	Vegetable oil–alcohol	Fe_2_O_3_	150 ppm	BB Fe_2_O_3_150 ppm	1% increase in thermal efficiency 60% reduction of emission characteristics, reduction of NO_*x*_, CO, HC and smoke Better mixing Presence of secondary atomization, disruption of primary droplet Complete combustion	[57]
Biodiesel	*Azadirachta indica*	CaCO_3_ nano-fluids	3 mg/l 5 mg/l	B100CaCO_3_	Reduction of 4.08% specific fuel consumption reduction 3.9% increase of brake thermal efficiency 8.57% higher mechanical efficiency Reduction of NO_*x*_ and HC emission	[130]
Biodiesel	*Linum usitatissimum*	CuO	80 ppm 40 μmol/L 80 μmol/L 120 μmol/L	B20CuO80 ppm	Significant increase in thermal efficiency 3–4% increase of brake thermal efficiency 25% reduction of CO Reduction of NO_*x*_ and HC emission	[62]
Biodiesel–castoroil–diesel–bioethanol	Vegetable oil–*Ricinus communis* oil Vegetable oil–alcohol	CeO_2_-CNT	25 ppm 50 ppm 100 ppm	–	Reduction of HC, CO, CO_2_, smoke and NO_*x*_ Increase of calorific value and brake thermal efficiency	[84]
Biodiesel		FeCl_3_	20 μmol/l	BFeCl_3_25	Reduction of HC, CO, CO_2_, smoke and NO_*x*_ Increase of calorific value and brake thermal efficiency	[84]

## Future applications of nano-additives for microalgae-biofuel

Nano-additive application on microalgae-biofuel enhancement has been categorized into several stages from raw material production to end-product implications. The stages are:

(i) nano-additives for microalgae cultivation;
(ii) nano-additives for microalgae biomass conversion to biofuels;
(iii) nano-additives for microalgae-biofuel applications.

### Nano-additives for microalgae cultivation

Improvement of the microalgae biomass productivity with the minimum area requirement is considered as the main purpose of nano-additive application in the microalgae culture. Nano-technology is being applied for enzyme immobilization, since nano-structures broaden the immobilization surface area causing high loading power of enzymes and stability of immobilized enzymes. Enzyme immobilization can be performed in different approaches such as electrospun nanofibers, covalently attached enzymes into nano-fibres, and enzyme aggregate coatings on nanofibers. The enzyme immobilization was investigated on various carbon nano-particles, e.g., graphene oxide (GO), multi-walled carbon nano-tubes (MWNTs), oxidized-MWNTs (O-MWNTs), and fullerene (C60). Among these nano-structures, O-MWNTs yielded the highest, and C60 yielded the lowest [21, 22]. Nano-particles’ (NPs’) application was implemented on several microalgae species harvesting and yielded outstanding in each phase of the application. Application of nano-particles on microalgae harvesting claimed 20–30% microalgae production cost in large-scale application [22]. Table 5 presents the harvesting efficiency of several microalgae species cultivated with various nano-particles. Nano-particles also boosted the light conversion efficiency in photobioreactor (PBR) during the microalgae culture period. It is also worth mentioning that PBR is run by artificial light sources consuming additional energy and cost. However, during biomass growth, light sources do not reach in culture broth inadequately due to self-shading and biofilm formation on the PBR surface. To achieve desired illumination properties and photo-conversion efficiency in the PBR, various light-emitting diodes (LEDs) equipped with nano-materials fabrication are being implemented recently. Gallium aluminum arsenide (GAA)-fabricated LEDs have been experimented on laboratory scale algae culture so far. It was evident that the application of optical fibres in algal culture saves much energy, additional light cost, and increase efficiency [18]. Another latest development of nano-particle, integration of metallic nano-particles (MNPs) with localized surface plasmon resonance (LSPRs) amplifies the light scattering at certain wavelength [65]. An experimental study revealed that silver nano-particles’ (Ag-NPs’) suspension in plasmonic mini-PBRs backscatter blue light strongly. The blue light increased the photosynthetic efficiency significantly for green microalgae, *Chlamydomonas reinhardtii,* and blue–green microalgae, *Cyanothece* 51142, and 30% higher microalgae biomass have been obtained [48]. Nano-particles addition in microalgae cultivation can also improve the yield of the CO_2_ absorption from the atmosphere and CO_2_ sequestration that can boost the biomass growth. For instance, nano-bubbles in microalgae culture remained stable for a longer time. Nano-bubbles also floated algae biomass into the culture, ensured high mass transfer efficiency, and improved biomass density by sufficient accumulation of CO_2_, O_2_ stripping, and minor buoyancy. Moreover, nano-bubbles suspended the biomass around airlift-loop bioreactor (ALB) and required less energy than micro-bubbles. Uniform nonporous membrane of ALB was also capable of producing 100 nm sized bubbles for this purpose [18, 66, 67]. The previous studies also delineated that nano-additives played a significant role in flocculation and separation process before biofuel production besides microalgae harvesting [22].

**Table 5:** Harvesting efficiency of various microalgae species with the addition of nano-particles

Microalgae species	Nano-particles	Harvesting efficiency (%)	References
*Chlorella ellipsoidea*	Modified Chu 13, doses 380 mg/g	90	[22]
*Chlorella ellipsoidea*	Fe_3_O_4_ nano-particles functionally coated with polyethylenimine (PEI)	97	[22]
*Chlorella ellipsoidea*	Iron oxide and cationic polyacrylamide (CPAM), doses 120 mg/l	> 95	[131]
*Botryococcus braunii*	Modified Chu 13, doses 20 mg/g	100	[22]
*Botryococcus braunii*	Iron oxide and CPAM, doses 15 mg/l	> 95	[131]
Marine *Nannochloropsis* sp.	Magnetic Fe_3_O_4_ nano-particles, doses 99 mg/g	95	[22]
*Chlorella* sp.	Surface-functionalized iron-oxide nano-particles (SF-IONPs) with PDA (poly dimethylammonium chloride)	99	[22]
*Chlorella* sp.	Chitosan/magnetic nano-particles (CS-MNPs)	97	[132]
*Chlorella* sp. KR-1	Naked Fe_3_O_4_ magnetic particles	90	[22]

### Nano-additives for microalgae biomass conversion to biofuels

Among microalgae biofuel, biodiesel has been appeared as the most popular and commercial biofuel in the mobile fuel market. For the case of biodiesel production, applications of acidic and basic nano-catalyst spheres can substitute the chemical compounds such as sodium methoxide by reacting with the free fatty acids and oils. Additional advantages of these nano-catalysts are recyclability and positive economical impact. Moreover, reactions can take place with low temperature and pressure as well as this approach reduces the contaminant release to the environment borne by sodium methoxide [6]. An industrial biodiesel study demonstrated that commercial CaO-NPs presented 91% biodiesel conversion efficiency during scaled-up catalytic transesterification [18]. Experimental study of microalgae cultivation with spherical nano-particles composed with sand (silica) and calcium compounds revealed that microalgae cellular growth increased drastically without harming harvesting as well as biofuel production from vegetable oil. The best way to address this issue was described as one of the major driving factors for commercial biofuel, biofuel production cost dropped effectively [6–8, 68]. The experimental study mentioned that mesoporous silica nano-catalyst, Ti-loaded SBA-15 presented ten times higher free fatty acids (FFA) and water tolerance level than any other catalysts for biodiesel production from vegetable oil as well as this nano-catalyst performed three times better than other effective nano-catalysts titanium silicalite-1 (TS-1) and titanium dioxide silicate (TiO_2_-S) [69].

Moreover, Ti-loaded Santa Barbara Amorphous-15 (SBA-15) nano-catalyst application reduced the chemical (alkaline catalyst, NaOH) cost of transesterification process for biodiesel production by recycling the nano-catalyst as well as this process is more environment-friendly [6, 69]. On the other hand, sulfate incorporated Ti-SBA-15 also performed as biocatalyst to convert vegetable oil to 100% esterified bio-lubricant. In consequences, this nano-particle can be expected to produce bio-lubricant from bio-oil of microalgae [70]. Other study showed that Niobia (N_2_O_5_) incorporated with SBA-15 application on biodiesel production from biomass through esterification presented a significant scenario for microalgae-biodiesel yield [71]. Another study delineated that the enzyme extracted from *Pseudomonas cepacia* conjuncts with the nano-particles such as polyacrylonitrile (PAN) nanofibre, Fe_3_O_4,_ and nanoporous gold; silica nano-particles with lipase enzyme from *Rhizopus miehei*; ferric silica and magnetic nano-particles with lipase from *Burkholderia* sp., polyacrylonitrile nano-fibre bound with lipase from *Thermomyces lanuginosa* has performed very effectively to produce biodiesel from various bio-oil by the transesterification process [7]. Furthermore, nano-magnetic biocatalyst of KF/CaO–Fe_3_O_4_, Li(lithium)-doped CaO, Fe_2_O_3_–CaO, sulfate (SO_4_^−^) incorporated Zi (zirconium), sodium titanate and carbon-based nano-tubes and nano-particles reached up to 95% or above biodiesel yield from diverse types of biomass and biodegradable waste [7, 72]. Besides enhancement of biodiesel-yield efficiency, a type of NP, zeolite (an alumina silicate mineral), has been used as commercial absorbent during the transesterification process. Zeolites absorbed the undesirable moisture content (4–6%) and produced pure glycerine as co-product besides biodiesel. Mesoporous nano-particles also presented a vital capability for continuous microalgal-biofuel generation from biomass without cell lysis. Zeolites also removed lipids from the microalgae cell membrane [18, 73]. Table 6 presented the applications of nano-additives for biodiesel-yield enhancements during microalgae to biofuel conversion, suitable conversion processes, and efficiencies.

**Table 6:** Applications of nano-additives for biodiesel-yield enhancements during microalgae to biofuel conversion, suitable conversion processes, and efficiencies

Nano-additives	Conversion processes	Conversion efficiency	References
Calcium oxide nano-particles’ blends (CaO-NPs)	Catalytic transesterification	91%	[18]
Mesoporous silica nano-catalyst, Ti-loaded SBA-15	Transesterification	10 times higher yield than other catalysts 3 times higher than other effective nano-catalysts TS-1 and TiO_2_–S	[6, 69]
Niobia (N_2_O_5_) incorporated with SBA-15	Esterification	Significant increase of biodiesel yield	[71]
PAN nanofibre, Fe_3_O_4_ and nanoporous gold incorporation, silica nano-particles, ferric silica and magnetic nano-particles incorporation, polyacrylonitrile nano fibre transesterification process	Transesterification	Effective rise in biodiesel productivity	[7]
KF/CaO–Fe_3_O_4_, Li(lithium)-doped CaO, Fe_2_O_3_–CaO, sulfate (SO_4_^−^) incorporated Zi (zirconium), sodium titanate and carbon-based nano-tubes and nano-particles	Transesterification	≥ 95%	[7, 72]

Nano-particles were efficiently capable to perform as immobilizing beds for valuable enzymes due to their large surface area-to-volume ratio. This capability of NPs broke down the long chains of complex sugar of microalgae, converted it to simple sugar, and consequently turned into bioethanol via the fermentation process. Due to the large surface area, the interaction between the surface of the nano-particles and fuel surrounded by them achieved adequate stability to overcome density variations. Nano-particles prepared by carbon nano-tubes doped with iron-oxide nano-particles presented excellent biocatalytic efficiency in a bioreactor, recyclable option enzyme applications, less capital cost as well as better enzyme loading for this purpose [6, 7, 74]. A catalytic study mentioned that mesoporous niobium oxide (N_2_O_5_) application on complex sugar (sucrose) possessed both Lewis acid (LA) and Bronsted acid (BA) sites to convert fructose to 5-hydroxymethylfurfural (HMF) with the highest yield so far. The synergistic catalytic effect from a large amount of both LA and BA acid site quantities and surface areas played a positive impact on the reaction rate with a few times faster conversions [75]. Functionalised multiwall carbon-nano-tube (MWCNT) immobilization presented more than 55% initial activity of microalgal hydrolysis for *Candida Antarctica.* Nano-catalysts such as cobalt–molybdenum fabricated with Si/Al have been experimented on *Botryococcus braunii* and presented stable hydrocarbons. Another nano-catalyst, mobil composition of matter No. 41 (MCM-41), mesoporous material effectively reduced oxygenated fractions of bio-oil through catalytic pyrolysis [18].

Nano-catalysts can synthesize biomethane produced from microalgae from wastewater into pure hydrogen and carbon content. In a further step, this methane can produce biogas through anaerobic digestion. Biogas could be used as raw material of bio-fuelled electricity generation further. The elemental carbon can also be utilized as pure nano-graphite for the applications on batteries, aerospace, automobiles, and others [6]. The latest development conducted by quantum sphere on marine microalgae species evinced biogasification from wet microalgae biomass by metal nano-catalysts [18]. Besides that, nano-particles such as TiO_2_, CeO_2_ were manifested to improve 10–11% of the biogas yield from wastewater treatment. Therefore, these nano-particles can be projected further for the biomethane production from microalgae grown in wastewater [7]. Apart from that, nano-substances with SiO_2_, nano-particles of platinum (Pt), nickel (Ni), cobalt (Co), and iron (Fe) can increase methane production from biomass up to 70%. Nano-fly ash and nano-bottom ash were proved to increase biomethane yield up to 3.5 times more. Nano-metal oxides, e.g., MgO, CaO, and SrO, nano-materials such as silica, single-walled nano-tubes of carbon-based materials, nano-clay, and nano-zero valence metal applications in biodiesel, bio-hydrogen, and biomethane production from microalgae and other biomass presented outstanding yield. These nano-particles can be projected for large-scale microalgal-biofuel production in the future to obtain revolutionary yield [7, 8]. In addition, nano-hybrid catalysts are being commercialized as emulsion stabilizers in industrial applications. For instance, quaternary ammonium salts have been documented as an emulsifying surfactant for separation, extraction, isolation, and purification of biofuels. Carbon nano-tubes with silica fusion, SiO_2_–MgO nanohybrids have been performed as stabilizers on bio-oil in water emulsion due to its inherent hydrophobicity and resulted in full conversion in different emulsion phases [18].

### Nano-additives for microalgae-biofuel applications

Solid nano-particles, nano-fluids, or nano-droplets with biofuel and fossil fuel were proved to improve the fuel lubricity, cetane number, burning rate, chemical reaction, catalytic performance, fire/flash point, heat and mass transfer efficiency and water co-solvency as well as decrease delay period [76, 77]. That resulted in more complete and cleaner combustion of microalgae biofuel mixed with fossil fuel in compression ignition (CI), spark ignition (SI), and direct ignition (DI) engines. In line with that, nano-technology applications showed the capability of amplifying microalgal-biofuel combustion efficiency and reduced soot, NO_*x*_, smoke, HC, CO_2,_ and CO emission to the environment up to 72% [6, 76, 78]. Application of solid nano-additives such as alumina (Al_2_O_3_), CERIA, carbon nano-tubes (CNT), Co_3_O_4_, ZrO_2_, La_2_O_3_, CeO_2_, SiO_2_, Ni_2_O, TiO_2_, ZnO, Fe_2_O_3_, CuO, Ce_*x*_Zr_(1–*x*)_O_2_, and amide-doped MWCNTs-CeO_2_ boosted the engine power, torque, and brake thermal performance of biodiesel (extracted from microalgae and other biomass) in CI and DI engines up to 11% [59, 76, 79, 80]. The experimental study of nano-particles on DI engines demonstrated that nano-particles blended with biodiesel as well as diesel–biodiesel mixture performed outstanding. The effectiveness was higher compared to usual catalysts [61, 81]. Another study presented that nano-particles of CeO_2_ incorporated with an emulsion of biofuel with sol–gel combustion technology performed excellent mono-cylinder 4 stroke direct CI and DI engines without any hardware modification. Nano-particles addition with biofuels escalated the fuel calorific value, fastened evaporation rate, improved brake-specific fuel consumptions and thermal efficiency, reduced greenhouse gases (GHGs) such as CO, NO_*x*_, and smoke, and unburned HCs. Chemical reactions between CeO_2_ and GHGs gases are presented in Rc. 2, Rc. 3, and Rc. 4 [82]:

2 $$\left( {2y + x} \right){\text{CeO}}_{2} + {\text{H}}_{x} {\text{C}}_{y} \to \left[ {\frac{2y + x}{2}} \right]{\text{Ce}}_{ 2} {\text{O}}_{ 3} + \frac{x}{2}{\text{H}}_{ 2} {\text{O}} + \frac{y}{2}{\text{CO}}_{ 2}$$

3 $$2{\text{CeO}}_{ 2} + {\text{CO}} \to {\text{Ce}}_{ 2} {\text{O}}_{ 3} + {\text{CO}}_{ 2}$$

4 $${\text{Ce}}_{ 2} {\text{O}}_{ 3} + {\text{NO}} \to 2{\text{CeO}}_{ 2} + \frac{1}{2}{\text{N}}_{ 2} .$$

In contrast, liquid nano-additive, nano-Al-droplet application (nano-suspension) on biofuel mixture has been manifested more efficient than even micro-suspension. Liquid nano-additives also presented outstanding performance by achieving better suspension than *n*-decane-based fuels. Nano-Al suspension with ethanol was strong enough for a longer period than other particles, because ethanol tended to form a gel around the nano-particles due to higher viscosity [74]. Nano-droplets coated a monolayer on the mechanical parts of the engine touched with liquid fuel and improved fuel efficiency [18]. In addition, NPs such as nano-Al, Al_2_O_3_, CuO, MgO, MnO, and ZnO incorporated with water–diesel–biodiesel (E10) emulsion and bioethanol performed remarkably. Among these NPs, Al_2_O_3_ performed the best because of mandate disabling, consistent torque boosting, higher heat of combustion, super-high DTG_max_ value, tiniest size of water droplets, the minimum value of brake-specific fuel consumption, and lowest values of Soot, NO_*x*_, CO, and HC [19, 83].

### Challenges and future prospects

Although nano-additive applications played significant role in microalgae cultivation, harvesting, conversion to biofuel and biofuel applications to enhance the efficiency, yet some challenges remained before the implementation of nano-additives for the mercantile approach. Most of the nano-additives from experimental research were not well-characterized in terms of particle size, shape, and size distribution as well as clustering [84]. Before large-scale application, well characterization of nano-particles and nano-fluids must be studied comprehensively. Appropriate nano-additive selection, preparation methods, and time for the selected application should be emphasized for optimum productivity. The effect of nano-catalyst implementation for microalgae-biofuel combustion quality, engine performance, and gas emission should be well studied and well-understood before implementation. In line with that, the availability of appropriate nano-additives with large amount might be a challenge for mass application though for laboratory scale, nano-additives are adequately available. Another constraint is cost-effectiveness of nano-catalysts for an industrial application which may hinder the commercial perspective, since many nano-catalysts are quite expensive.

Along with the potential microalgae determination and biofuel generation, integration of a plant design of value-added co-products will be the predominant advantage of the overall project with the economical aspect. This review encouraged biofuel research and development (R&D) sector worldwide to convert their unused, abandoned and wastewater sources, wet, and barren lands into microalgae farm as an eminent source of biofuel production. However, it should be highlighted that based on the existing research experiments, microalgae fuel production still stands at initial stage due to downward economic profile worldwide. Nano-additive applications on microalgal biofuel are yet confined into laboratory and pilot scale which can be counted as a significant limitation. Hence, it is strongly recommended to figure out large-scale process development with nano-additive applications for enhancement of microalgal growth, biofuel transformation processes, and fuel utilization in CI and DI engines. Nano-additive applications at different stages from microalgae culture to end-product utilization have a strong possibility to gain economical feasibility. Therefore, the detailed techno-economic analysis must be commanded to determine whether NP applications on microalgae biofuel are economically favorable or not, since economical issue is one of the most effective factors behind large-scale plant setup. Besides, these applications also have positive impact on the environment with value-added co-product generation into near further. Since the nano-additive utilization manifested itself environment-friendly, still a comprehensive life cycle assessment should be conducted to present the environmental positivities transparently. Besides all these factors, public safety, impact on flora and fauna, and the possibility of bio-hazards are also needed to be analysed extensively before commercialization.

## Conclusions

Microalgae utilization for biofuel production is undoubtedly desirable all over the world. Though this approach is energy-efficient and environment-friendly, experts are still looking for an innovation that can boost the microalgae-biofuel yield from primary stage to end product as well as shift the whole process towards a cost-effective fuel solution. Hence, this review was emphasized on the synergistic effect of nano-additive-enhanced microalgal biofuel for mercantile approach and fuel-yield extension. Application of various forms of nano-additives in different phases on microalgae growth to biofuel demonstrated an excellent outcome that may project revolutionary improvement of commercial microalgae biofuel. However, the sustainability analysis of stepwise production rounds for microalgae biofuel still presented a bare need of further research and innovative concepts. These concepts may determine the most appropriate nano-additive for the desired type of biofuel in the context of economical aspect. Since nano-additive application on microalgae is quite new research concept, policy making and implementation of nano-additives will remain as the most vital issues for commercial output especially in developing countries. Therefore, managerial insights are needed to be emphasized further on proper policy, socio-economic impact, advantages and limitations for the overall system to attract the government and non-government fuel industries.

### Highlights

- Enhancement of microalgae cultivation and harvesting by nano-bubbles and nano-particles application.
- Identification of suitable microalgae species, possible biofuels from microalgae, latest conversion technologies, processes, and required equipments.
- Excellent microalgal-biofuel yield by nano-droplet and nano-additives.
- Complete and cleaner combustion in fuel engines by nano-emulsion and nano-stabilizers.

## Data Availability

Not applicable.

## Abbreviations

ABE: acetone–butanol–ethanol
Ag-NPs: silver nano-particles
Ag_2_O: silver oxide
ALB: airlift-loop bioreactor
Al_2_O_3_: aluminum oxide/alumina
Al-droplet: aluminum droplet
BA: bronsted acid
C60: fullerene
CaCO_3_: calcium carbonate
CeO_2_/CERIA: cerium oxide
CaO: calcium oxide
CaO-NPs: calcium oxide nano-particles blends
CI: compression ignition
CO_2_: carbon-dioxide
CO: carbon mono oxide
Co_3_O_4_: cobalt oxide
CNT: carbon nano-tubes
CuO: copper oxide
Ce_*x*_Zr_(1−*x*)_O_2_: cerium–zirconium oxide
CS-MNPs: chitosan/magnetic nano-particles
*D*: direct ignition
DES: deep eutectic solvent
DTG_max_: maximum derivative thermogravimetry
FF: free fatty acids
Fe_2_O_3_: ferric oxide
FeCl_3_: ferric chloride
GAA: gallium aluminum arsenide
GHGs: greenhouse gases
G: graphene oxide
HC: hydro-carbon
HMF: 5-hydroxymethyl furfural
LA: Lewis acid
La_2_O_3_: lanthanum oxide
LEDs: light-emitting diodes
LSPRs: localized surface plasmon resonance
MWCNTs: multiwall carbon nano-tubes
MCM-41: mobil composition of matter No 41
MgO: magnesium oxide
MnO: manganese oxide
MNPs: metal nano-particles
N_2_O_5_: niobia/niobium oxide
Ni_2_O: nickel oxide
NO_*x*_: nitrogen oxide
O-MWNTs: oxidized MWNTs
PAN: polyacrylonitrile
PBR: photobioreactor
PDA: poly dimethylammonium chloride
R&D: research and development
Rh_2_O_3_: rhodium oxide
SBA-15: santa barbara amorphous-15
SCWG: supercritical water gasification (SCWG)
SF-IONPs: surface-functionalized iron-oxide nano-particles
SI: spark ignition
SiO_2_: silicon dioxide
SSCF: simultaneous saccharification and co-fermentation
SHCF: separate hydrolysis and co-fermentation
SHF: separate hydrolysis and fermentation
SSF: simultaneous saccharification and fermentation
SrO: strontium oxide
TiO_2_: titanium dioxide
TS-1: titanium silicalite-1
TiO_2_-S: titanium dioxide silicate
Zeolite: alumino silicate mineral
ZnO: zinc-oxide
ZrO_2_: zirconium dioxide
